# Disrupted topological organization of functional brain networks in traumatic axonal injury

**DOI:** 10.1007/s11682-023-00832-z

**Published:** 2023-12-04

**Authors:** Jian Li, Yongqiang Shu, Liting Chen, Bo Wang, Linglong Chen, Jie Zhan, Hongmei Kuang, Guojin Xia, Fuqing Zhou, Honghan Gong, Xianjun Zeng

**Affiliations:** 1 Department of Radiology, The First Affiliated Hospital, Nanchang University, 17 Yongwai Zheng Street, Donghu District, Nanchang City, 330006 Jiangxi China; 2 Neuroimaging Lab, Jiangxi Province Medical Imaging Research Institute, Nanchang, China; 3 Medical Imaging Center, First Affiliated Hospital of Jinan University, Guangzhou, China

**Keywords:** Traumatic axonal injury, Traumatic brain injury, Brain functional networks, Graph theory, Resting-state functional magnetic resonance imaging

## Abstract

Traumatic axonal injury (TAI) may result in the disruption of brain functional networks and is strongly associated with cognitive impairment. However, the neural mechanisms affecting the neurocognitive function after TAI remain to be elucidated. We collected the resting-state functional magnetic resonance imaging data from 28 patients with TAI and 28 matched healthy controls. An automated anatomical labeling atlas was used to construct a functional brain connectome. We utilized a graph theoretical approach to investigate the alterations in global and regional network topologies, and network-based statistics analysis was utilized to localize the connected networks more precisely. The current study revealed that patients with TAI and healthy controls both showed a typical small-world topology of the functional brain networks. However, patients with TAI exhibited a significantly lower local efficiency compared to healthy controls, whereas no significant difference emerged in other small-world properties (Cp, Lp, γ, λ, and σ) and global efficiency. Moreover, patients with TAI exhibited aberrant nodal centralities in some regions, including the frontal lobes, parietal lobes, caudate nucleus, and cerebellum bilaterally, and right olfactory cortex. The network-based statistics results showed alterations in the long-distance functional connections in the subnetwork in patients with TAI, involving these brain regions with significantly altered nodal centralities. These alterations suggest that brain networks of individuals with TAI present aberrant topological attributes that are associated with cognitive impairment, which could be potential biomarkers for predicting cognitive dysfunction and help understanding the neuropathological mechanisms in patients with TAI.

**Supplementary Information:**

The online version contains supplementary material available at 10.1007/s11682-023-00832-z.

## Introduction

Traumatic axonal injury (TAI) is often diffuse after traumatic brain injury (TBI) and frequently leads to disruption of brain networks, which is recognized as a major cause of poor functional outcomes, including cognitive disorder, motor deficits, behavioral problems, and emotional lability, comprising a “disconnection syndrome”. (Bonnelle et al., 2011; Castellanos et al., 2011; Fagerholm et al., 2015; Ponsford et al., 2014; Sharp et al., 2014). TAI is characterized by widespread axonal damage caused by shearing forces of rotational acceleration and deceleration occurring at the time of injury. The recent development of new neuroimaging techniques has substantially improved our understanding of the underlying pathophysiology of persistent cognitive impairment after TBI by allowing the visualization of local changes in brain structure and function networks (Li et al., 2023; Pan et al., 2023; Sharp et al., 2014). Unlike focal injuries that affect specific brain regions, axonal injury fundamentally impairs the substrate through which brain networks communicate with each other. TAI disrupts the integrity of white matter microstructure, which affects brain functional connectivity supporting cognitive function (Kinnunen et al., 2011). The change in functional connectivity can be attributed to the presence of diffuse white matter pathology, as well as reductions in both gray and white matter volume (Caeyenberghs et al., 2017). Since the patterns of TAI are generally widespread and highly variable among individuals (Hellyer et al., 2013; Jolly et al., 2021; Kinnunen et al., 2011), this condition may have complex effects on brain network function after TBI. Previous studies showed that TAI primarily damages the long-distance white matter pathways connecting the nodes of large-scale distributed brain networks, resulting in a reduced capacity to integrate information between different brain regions (Bonnelle et al., 2011; Fagerholm et al., 2015; Kinnunen et al., 2011; Pandit et al., 2013; Sharp et al., 2014). Therefore, understanding the effects of TAI on brain function and behavior requires a detailed investigation on the global brain network function. However, the characteristics of information flow within and between regions remain largely unknown, as well as the effect of insult-induced alterations on the interactions between the functional network nodes in patients with TAI.

Functional connectivity networks are primarily concerned with the connective properties of temporal coherences between blood oxygen level-dependent functional magnetic resonance imaging (fMRI) signals from both local and distant brain regions (Salvador et al., 2005), and connectivity patterns can also be represented graphically. Graph theory is a unique and powerful tool to quantify the topological features of functional networks. In graph theory, a network consists of a set of ‘nodes’, which represent cortical and sub-cortical anatomical regions, and ‘edges’, the properties of the connections between these nodes (e.g. white matter fibers). This schematic structure reflects the network connectivity and offers an integrative approach to explore the communication and transmission of information between regions in the whole brain by investigating the different properties of the cerebral networks, including integration, segregation, centrality, and small-world properties. An increasing number of studies used graph theory to investigate the effects on brain functional networks of several neurological diseases, such as Alzheimer's disease (John et al., 2017), autism spectrum disorder (Chen et al., 2021), schizophrenia (Liu et al., 2016), and functional dyspepsia (Zhang et al., 2022). A recent study reported that patients with cerebral small vessel disease and microbleeds exhibited significantly reduced clustering coefficient, global efficiency, and local efficiency, and an increased shortest-path length, compared to controls and patients with the same disease and no cerebral microbleeds. This finding indicates a disrupted balance between local specialization and global integration in functional connectivity networks. Similarly, functional connectivity alterations associated with TBI have recently been studied using the mathematical concepts of graph theory (Boroda et al., 2021; Fagerholm et al., 2015; Kim et al., 2022; Li et al., 2023; Pandit et al., 2013; Raizman et al., 2020). However, the findings in these studies were contradictory and inconsistent due to the heterogeneity of patients with TBI, with large differences in severity, etiology, type of injury and mechanism, as well as recovery rate and burden of chronic symptoms (Maas, 2016; O'Brien et al., 2020; Caeyenberghs et al., 2017).

Graph theoretical analysis has already offered precious insights into the dysfunction of brain networks following TBI. For example, patients with moderate-to-severe TBI showed higher degree and strength of connectivity and higher values of local efficiency in the late phase of the disease (averaged 4.83 years prior to the study) compared to healthy controls (HCs), whereas these differences were significantly correlated with poorer switching task performance (Caeyenberghs et al., 2012). In contrast, a reduction in network efficiency was observed in patients with TBI in other studies (Han et al., 2016; Pandit et al., 2013), and Han et al. (2016) indicated that disruptions in nodal connectivity predominantly appeared in the inter-network edges investigated, rather than the intra-network edges, as noted with a network-based statistics (NBS) analysis. Alterations in graph metrics, such as higher connectivity degree, clustering coefficient and strength, fewer inter-module connections, and reduced network efficiency, indicated that the functional connectivity network was significantly disrupted in patients with TBI, might be associated with hyperconnectivity and a suboptimal global integration (Caeyenberghs et al., 2017). These previous studies on TBI have shown that global changes in network metrics are accompanied with alterations in nodal metrics in specific brain regions (Caeyenberghs et al., 2012; Messe et al., 2013; Pandit et al., 2013). The ratio between global and local connectivity can be quantified by the concept of small-worldness, reflecting the requirement for the networks to satisfy the opposing demands of local and global processing (Kaiser & Hilgetag, 2006). However, the small-world attributes had been quantified by graph theory to assess the functional connectivity after TBI with mixed results (Caeyenberghs et al., 2012; Hillary et al., 2014; Kuceyeski et al., 2016). The inconsistent findings suggest that both global integration and regional segregation of the brain networks can be affected after TBI. However, how TBI disrupts the topological organization of the brain connectivity networks still remains to be elucidated. Moreover, the underlying mechanisms connecting the pathophysiology of TBI and the topological features of functional networks are still unclear. This uncertainty may be due to the problematic nature of the index (Rubinov & Sporns, 2010), heterogeneity in patient populations (including diffuse and focal lesions and at different stages of the disease), and parcellation schemes in most patients with TBI reported in previous studies (Caeyenberghs et al., 2017). Therefore, we recognize that variability is a hallmark of TBI, and subgroup analyses should be performed. A feasible approach to counter the issue of heterogeneity is to select relatively homogeneous individuals such as “pure” TAI patients (Caeyenberghs et al., 2017; Turner et al., 2011), investigating the extent to which graph metrics are preserved or affected.

The purpose of this study was to examine the changes in whole-brain functional networks in patients with relatively “pure” TAI, characterizing the global and local topological properties with graph theory and NBS analyses. We hypothesized that the topological organization of brain functional networks is disrupted in patients with TAI, and that these alterations may be related to impairments in functional connectivity and cognitive function. In particular, we aimed to construct a functional connectome in patients with TAI and determine whether these patients show the following: (1) altered global and regional properties in whole-brain functional networks, (2) disrupted functional connectivity in specific subnetworks, and (3) possible relationships between global and local topological properties and cognitive disorders or clinical measures.

## Materials and methods

### Participants

Twenty-eight patients with TAI (20 males, 8 females; mean age: 38.96 ± 14.26 years) were retrospectively selected from a TBI database (*n* = 182) including multimodal MRI images of patients recruited from the Department of Neurosurgery of the First Affiliated Hospital of Nanchang University between October 2013 and February 2017. The cause of TAI was motor vehicle accident for all patients selected. The following inclusion criteria were applied, as in a previous study (Li et al., 2017): 1) closed-head TBI via a mechanism consistent with TAI (i.e.,. high-velocity rotational or acceleration-deceleration forces); 2) hemodynamical stability to ensure a safe transfer to the scanner; and 3) patient age between 18–60 years. The exclusion criteria were as follows: 1) previous history of TBI or neurological disorders; 2) presence of any focal, mixed, or high-density lesion (including contusions, extra-axial hematomas, and/or intraparenchymal hemorrhages), with a volume > 1 mL, visible on head computed tomography (CT); 3) absent pupillary responses bilaterally; 4) any condition that could result in atypical MRI findings and compromised cognitive functions (i.e.,. prior brain tumor, epilepsy, multiple sclerosis, psychiatric disorders); and 5) any contraindication to MRI, including metal and/or electronic implants, claustrophobia, and possible or verified pregnancy.

Traumatic microbleeds are visible on susceptibility-weighted imaging (SWI) with clear margins and high sensitivity and are often considered a marker of TAI. All patients selected exhibited subcortical traumatic microbleeds, as determined by a senior neuroradiologist. The vast majority of these typical microbleeds in patients with TAI have been described in our previous study (Li et al., 2017).

In addition, 28 healthy volunteers (20 males, 8 females; mean age: 38.59 ± 13.20 years), matched for age, sex, and education level and with no history of neurological or psychiatric disorders, were recruited as HCs. Informed consent was obtained from all the participants. This study was approved by the Institutional Review Board of the First Affiliated Hospital of Nanchang University and conducted in accordance with the principles of the Declaration of Helsinki.

### Clinical assessments

Each patient with TAI was assessed via a detailed clinical interview and several rating scales, mainly the Glasgow Coma Scale (GCS) to assesses the level of consciousness impairment and the Mini-Mental State Examination (MMSE) to evaluate the cognitive function. In addition, we assessed the patients with the Disability Rating Scale (DRS), Motor Assessment Scale (MAS), Adaptive Behavior Scale (ABS), Hamilton Anxiety Scale (HAMA), and Activity of Daily Living Scale (ADL), in the 2 h before the MRI scans.

### MRI data acquisition and preprocessing

All subjects underwent MRI scanning with a Trio 3.0 Tesla MRI system equipped with an 8-channel phased-array head coil (Siemens, Erlangen, Germany). Foam pads and earplugs were used to reduce involuntary head movements and external noise. The scanning protocol included the following sequences: (1) localizer, (2) T1-weighted MPRAGE structural images (176 sagittal slices; thickness/gap = 1.0/0 mm, in-plane resolution = 256 × 256, field of view (FOV) = 240 mm × 240 mm, repetition time (TR) = 1,900 ms, echo time (TE) = 2.26 ms, flip angle = 15°), and (3) echo-planar imaging pulse sequence (30 axial slices; thickness/gap = 4.0/1.2 mm, in-plane resolution = 64 × 64, FOV = 200 mm × 200 mm, TR = 2,000 ms, TE = 30 ms, flip angle = 90°, 240 time points). During the scan, the participants were asked to remain still, awake and calm, with their eyes closed, and avoid thinking as much as possible. In addition, conventional T2-weighted images, T2 fluid-attenuated inversion recovery (FLAIR) images, SWI, and diffusion-weighted imaging (DWI) of the brain were acquired in each subject for the diagnosis.

All preprocessing was performed using the Data Processing & Analysis Assistant for Resting-State Brain Imaging (DPABI, http://rfmri.org/dpabi) and Statistical Parametric Mapping (SPM12, https://www.fil.ion.ucl.ac.uk/spm/software/spm12/) packages in MATLAB 2016a (MathWorks, Natick, MA, USA). The first 10 volumes of resting-state fMRI data were discarded, and the remaining 230 volumes acquired from each participant were corrected for the differences in slice acquisition times. The resulting images were then realigned to correct for small movements between scans. Images with head motion that exceeded a 2-mm translation or 2° rotation during functional imaging were excluded. Individual T1-weighted structural images were co-registered with the mean realigned echo-planar images. The transformed structural images were then segmented into gray matter, white matter, and cerebrospinal fluid and registered to the Montreal Neurological Institute (MNI) space using the Diffeomorphic Anatomical Registration Through Exponentiated Lie Algebra (DARTEL) toolbox. The same transformation parameters were applied to the functional images for spatial normalization, and the volumes were then resliced at a resolution of 3 × 3 × 3 mm. A nuisance linear regression was performed to reduce the effects of confounding factors, using as covariates the 24 head-motion parameters (Friston 24-parameter model), white matter, cerebrospinal fluid, and whole-brain signal. Finally, temporal filtering (0.01–0.1 Hz) of the time series was performed.

## Functional network construction

### Definition of the nodes and edges

Automated anatomical labeling (AAL) was used to define the brain nodes, dividing the whole brain into 116 cortical and subcortical regions of interest (Chen et al., 2021; Liu et al., 2015; Tzourio-Mazoyer et al., 2002). The averaged time series of all voxels in each region of interest was extracted to obtain a representative time series for each subject using the AAL template. The Pearson’s correlation coefficients between the regional mean time series of all possible pairs among the 116 brain regions were calculated as edges in the network, resulting in a 116 × 116 Pearson’s correlation matrix for each participant, then converted into binarized matrices to facilitate the network topology analysis. A Fisher’s r-to-z transformation was performed to translate the individual correlation maps into normalized z-score maps.

### Graph theory analysis

The properties of the brain functional networks in patients with TAI and HCs were examined at the global and regional (nodal) levels using the Graph-Theoretical Network Analysis (GRETNA) toolbox (http://www.nitrc.org/projects/gretna/) (Wang et al., 2015). The brain functional networks were modeled based on an unweighted and undirected method. We applied a sparsity (Sp) threshold to all correlation matrices over a wide range of Sp levels (from 0.05 to 0.50, using intervals of 0.01) to explore the between-group differences in the brain functional network organization in patients with TAI and HCs. We used the AUC to identify significant between-group differences comparing the global network metrics and nodal characteristics of the whole-brain functional networks between the TAI and HC groups. We examined several global network metrics, including the small-world properties (Watts & Strogatz, 1998), such as the clustering coefficient (Cp), characteristic path length (Lp), normalized clustering coefficient (γ), normalized characteristic path length (λ), and small-world-ness (σ), and the network efficiency parameters, i.e.,. global efficiency (E_glob_) and local efficiency (E_loc_). We also examined the nodal network metrics (nodal degree centrality, nodal betweenness centrality, nodal efficiency, nodal clustering coefficient, nodal local efficiency, and nodal shortest-path length). The descriptions of graph metrics investigated in the current study were given in Table 1.

**Table 1:** Descriptions of graph metrics examined in the current study

Graph metric	Descriptions
Global network metrics	
Clustering coefficient (Cp)	The average inter-connectedness of a node’s direct neighbors
Characteristic path length (Lp)	The average shortest path length between any pairs of nodes
Normalized clustering coefficient (γ)	The clustering coefficient compared to matched random networks
Normalized characteristic path length (λ)	The characteristic shortest path length compared to matched random networks
Small-worldness (σ)	The normalized clustering coefficient divided by the normalized characteristic shortest path length, which reflect the balance of global efficiency and local efficiency
Global efficiency (E_glob_)	The efficiency of information transfer through the entire graph
Local efficiency (E_loc_)	The average efficiency of information transfer over a node’s direct neighbors
Nodal network metrics	
Nodal degree centrality	Measure of the number of connections connected directly to a node in the graph
Nodal betweenness centrality	Fraction of all shortest paths in the network that pass through a given node, which reflect the influences of a node over information flow between other nodes
Nodal efficiency	The efficiency of information transfer over a node’s direct neighbors, indicating the efficiency of the parallel information transmission of the node in the network
Nodal clustering coefficient	The inter-connectedness of a node’s direct neighbors
Nodal local efficiency	The inverse of the shortest average path length in a subgraph comprising of node and its adjacent neighbors
Nodal shortest path length	Measurement of the functional integration of nodes in brain networks

A small-world network meets the following criteria: γ = Cp_real_/Cp_rand_ > 1, and λ = Lp_real_/Lp_rand_ ≈ 1, or σ = γ/λ > 1 (Watts & Strogatz, 1998).

### Statistical analysis

The AUC of each metric was calculated to determine the between-group differences in small-world properties, network efficiency, and nodal characteristics, for statistical comparison within the Sp range mentioned above, including as covariates age, educational level, and mean frame displacement. Demographic and clinical variables of the patients with TAI and HCs were compared using independent two-sample t-tests with SPSS 22.0 (IBM, Armonk, NY, USA). The significance level was set at *p* < 0.05.

Cerebral regions were considered altered nodal centralities in patients with TAI when they exhibited significant between-group differences in at least one of the six nodal centralities (nodal degree, nodal betweenness centrality, nodal efficiency, nodal clustering coefficient, nodal local efficiency, and nodal shortest-path length). An NBS analysis (http://www.nitrc.org/projects/nbs/) was performed on Fisher’s z-transformed correlation coefficients to examine the differences in functional connections between the TAI and HC groups (Liu et al., 2016; Zalesky et al., 2010). A subset of connection matrices was generated based on the altered nodes in the patients with TAI. We applied the NBS method to define a set of suprathreshold links among any connected components (threshold: T = 2.368, *p* < 0.01). The significance of each component was estimated using the nonparametric permutation method (10,000 permutations). Age, educational level, and mean frame displacement were used as covariates. Finally, a partial correlation analysis was performed to assess the relationships between network metrics and clinical variables and evaluate the clinical correlates of these network property changes in patients with TAI. *P* values < 0.05 were considered statistically significant.

## Results

### Demographic and clinical features

The TAI and HC groups were accurately matched in terms of sex (*p* > 0.99), age (*p* = 0.99), and educational level (*p* = 0.87). Significant alterations were observed in the TAI group as expressed by clinical scale scores, including GCS, MMSE, DRS, MAS, ABS, HAMA, and ADL (Table 2).

**Table 2:** Demographic and clinical features of participants

Characteristics	TAI (*n* = 28)	HCs (*n* = 28)	*p*-value
Sex (male/female)	20/8	20/8	> 0.99
Age (years)	38.96 ± 14.26	38.59 ± 13.20	0.99
Education (years)	7.18 ± 3.44	7.32 ± 3.10	0.87
Injury-to-MRI interval (days) (median, range)	22 (2–210)	n/a	n/a
Handedness (right), %	100	100	1.0
GCS (Scanning)	13.82 ± 2.31	15 ± 0	0.01
MMSE	19.82 ± 10.15	29.71 ± 0.46	< 0.01
DRS	9.32 ± 6.15	0 ± 0	< 0.01
MAS	35.82 ± 11.71	48 ± 0	< 0.01
ABS	31.21 ± 12.78	15.09 ± 1.38	< 0.01
HAMA	10.57 ± 6.65	1.43 ± 1.03	< 0.01
ADL	32.43 ± 14.45	14.36 ± 0.62	< 0.01

The data are presented as mean ± SD. *TAI* traumatic axonal injury, *HCs* healthy controls, *GCS* Glasgow coma scale, *MMSE* mini-mental state examination, *DRS* disability rating scale, *MAS* motor assessment scale, *ABS* agitated behavior scale, *HAMA* Hamilton anxiety scale, *ADL* activities of daily living. *P*-values were obtained using a two-tailed chi-squared test or a two-sample two-tailed t-test, as applicable

### Differences in global network measures

**Fig. 1:**
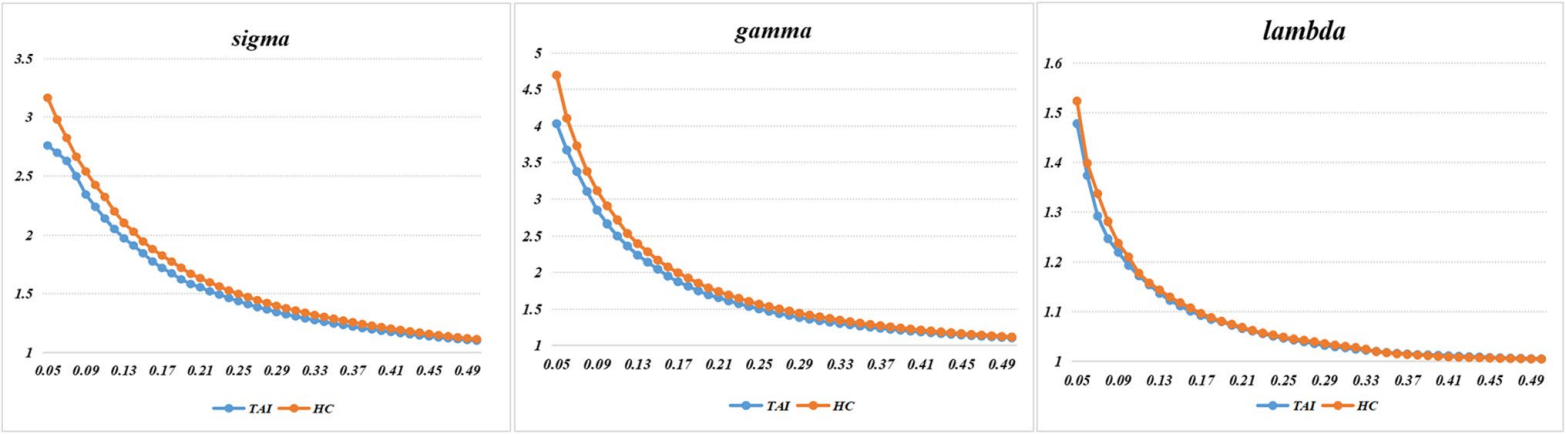
Small-world parameters of the brain functional network in patients with traumatic axonal injury (TAI) and healthy controls (HCs). The graphs show that in the wide range of thresholds examined (from 0.05 to 0.50), patients with TAI and HCs both exhibited normalized clustering coefficient (γ) obviously > 1, normalized path lengths (λ) approximately equal to 1, and small-world-ness (σ) > 1, suggesting that all participants show a typical small-world topology

**Fig. 2:**
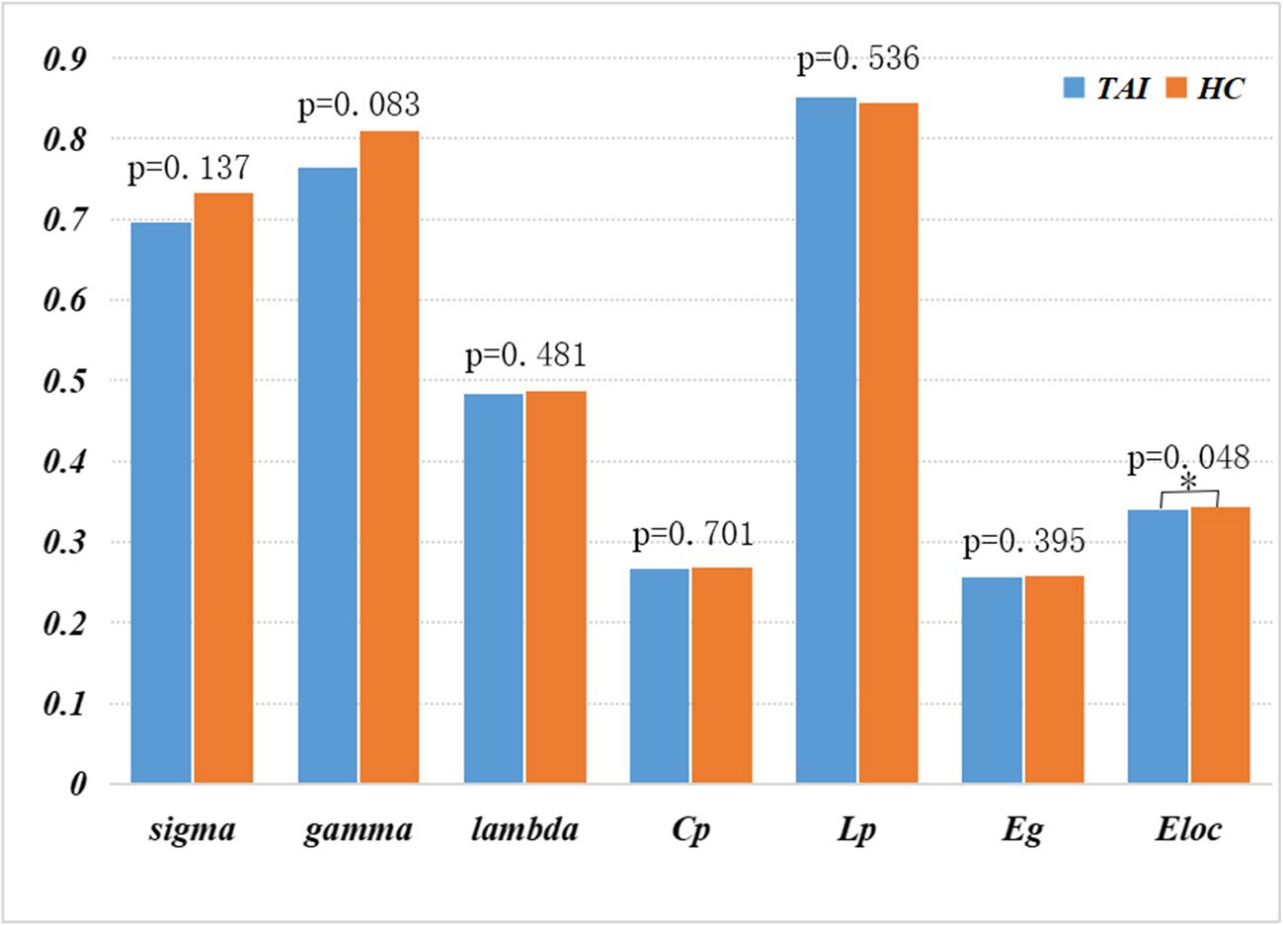
Graphs showing the small-world parameters and network efficiency of the whole-brain functional network in patients with traumatic axonal injury (TAI) and healthy controls (HCs). Patients with TAI showed a significantly lower E_loc_ (t =  − 2.026, *p* = 0.048). σ: small-world-ness; γ: normalized clustering coefficient; λ: normalized path lengths; Cp: clustering coefficient; Lp: characteristic path length; E_glob_: global efficiency; E_loc_: local efficiency

In the wide range of thresholds selected, patients with TAI and HCs both showed a typical small-world topology of the functional brain networks, expressed by a γ value > 1, λ value approximately equal to 1, and σ value > 1 (Fig. 1). Furthermore, the normalized clustering coefficient γ and the overall normalized path length λ were not significantly different between TAI and HC groups (t =  − 1.765, *p* = 0.083 and t =  − 0.709, *p* = 0.481, respectively), suggesting an overall preserved organization of the functional brain network in patients with TAI. However, these patients showed a significantly lower E_loc_ (t =  − 2.026, *p* = 0.048) compared to HCs. In contrast, there were no significant differences between groups in σ (t =  − 1.508, *p* = 0.137), Cp (t =  − 0.386, *p* = 0.701), Lp (t = 0.623, *p* = 0.536), or E_glob_ (t =  − 0.857, *p* = 0.395). The global network measures are illustrated in Fig. 2.

### Differences in regional network measures

Compared with HCs, patients with TAI showed several alterations in nodal centralities in the brain functional network (Supplementary Material 1), with significant between-group differences in at least one of the five nodal centralities (nodal betweenness, nodal degree, nodal clustering coefficient, nodal efficiency, and nodal local efficiency; *p* < 0.05, uncorrected), and none survived the correction for nodal shortest-path length. Patients with TAI showed significantly lower nodal centralities compared to HCs in the right olfactory cortex, left medial cingulate cortex, right superior occipital gyrus, left postcentral gyrus, right inferior parietal lobule, supramarginal gyrus and caudate nucleus bilaterally, left cerebellar hemisphere (VIII), and cerebellar vermis (10). Significantly higher nodal centralities were noted in patients with TAI in the left superior frontal gyrus, left superior orbital gyrus, superior medial gyrus bilaterally, right cerebellar hemisphere (crus 2, VII), and cerebellar vermis (4/5, 7) (*p* < 0.05, uncorrected). In addition, the patients with TAI exhibited some increased nodal centralities (nodal betweenness, nodal degree) and some decreased nodal centralities (nodal clustering coefficient, nodal local efficiency) in the right cerebellum (VI). These results suggest a modification in the local network integration following TAI.

### TAI-Related alterations in functional connectivity

**Fig. 3:**
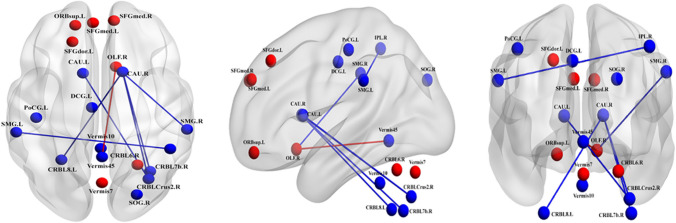
Altered functional connectivity within the network of disrupted areas significantly different between patients with traumatic axonal injury (TAI) and healthy controls (HCs). Abbreviations: SFGdor.L, left superior frontal gyrus; ORBsup.L, left superior orbital gyrus; SFGmed.L, left superior medial gyrus; SFGmed.R, right superior medial gyrus; CRBLCrus2.R, right cerebellum (Crus 2); CRBL7b.R, right cerebellum (VII); Vermis45, cerebellar vermis (4/5); Vermis7, cerebellar vermis (7); CRBL6.R, right cerebellum (VI); OLF.R, right olfactory cortex; DCG.L, left MCC; SOG.R, right superior occipital gyrus; PoCG.L, left postcentral gyrus; IPL.R, right inferior parietal lobule; SMG.L, left supramarginal gyrus; SMG.R, right supramarginal gyrus; CAU.L, left caudate nucleus; CAU.R, right caudate nucleus; CRBL8.L, left cerebellum (VIII); Vermis10, cerebellar vermis (10). The blue edges represent the reduced functional connectivity, and the red edges the higher functional connectivity in patients with TAI compared to HCs. Undirected edges correspond to t-values, with larger t-values represented by thicker edges (*p* < 0.01, uncorrected)

Using the NBS approach, we identified a disconnected functional subnetwork, with 20 nodes and six connections, that was significantly altered in the TAI group (*p* < 0.01, uncorrected). This subnetwork, comprising pairs of nodes, exhibited between-group differences in nodal centralities. Compared to HCs, patients with TAI exhibited significantly reduced functional connectivity between the right inferior parietal lobule and left supramarginal gyrus, right olfactory cortex and right supramarginal gyrus, both caudate nuclei and right cerebellar hemisphere (crus 2), and right caudate nucleus and right (VII) and left (VIII) sides of the cerebellum. Patients with TAI showed significantly higher functional connectivity between the right olfactory cortex and cerebellar vermis (4/5) compared to HCs. These connections mainly link regions over long distances (Table 3, Fig. 3).

**Table 3:** Alterations in functional connectivity between TAI and HCs

Brain region 1	Brain region 2	*t*-value	*p*-value
L Supramarginal Gyrus	R Inferior Parietal Lobule	− 2.760	0.008
R Supramarginal Gyrus	R Olfactory cortex	− 2.869	0.006
R Cerebellum (Crus 2)	L Caudate Nucleus	− 3.706	< 0.001
R Cerebellum (Crus 2)	R Caudate Nucleus	− 3.446	0.001
R Cerebellum (VII)	R Caudate Nucleus	− 3.955	< 0.001
L Cerebellum (VIII)	R Caudate Nucleus	− 3.538	0.001
Cerebellar Vermis (4/5)	R Olfactory cortex	3.059	0.003

Alterations in functional connectivity within the networks of disrupted areas significantly different between patients with TAI and HCs. (*p* < 0.01, uncorrected). *TAI* traumatic axonal injury, *HC* healthy controls, *L* left, *R* right.

### Correlations between network measures and clinical variables

**Fig. 4:**
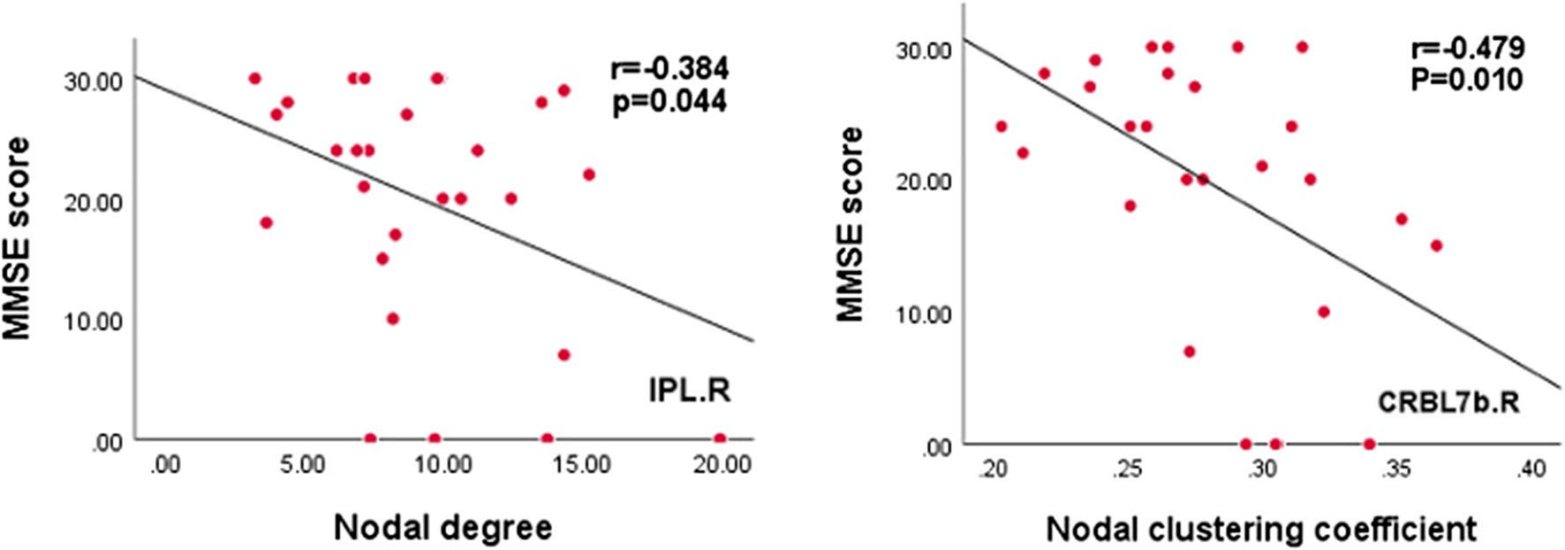
Relationships between altered nodal centralities and clinical measurements in patients with traumatic axonal injury (TAI). The nodal degree of the right inferior parietal lobule (IPL.R) and the nodal clustering coefficient of the right cerebellar hemisphere (VII) (CRBL7b.R) were negatively correlated with the Mini-Mental State Examination (MMSE) score. *P* values < 0.05 were considered statistically significant

The nodal degree of the right inferior parietal lobule and the nodal clustering coefficient of the right cerebellar hemisphere (VII) had significant negative correlations with the MMSE score (r =  − 0.384, *p* = 0.044, and r =  − 0.479, *p* = 0.010, respectively). Other topological network measures were not significantly correlated with clinical assessments (Fig. 4).

## Discussion

Our results can be summarized in five main findings: (1) all participants presented typical small-world attributes in the brain functional networks, with no differences between TAI and HC groups in small-world properties and global efficiency; (2) the patients with TAI exhibited lower E_loc_ in brain functional networks; (3) they also exhibited aberrant nodal centralities in some regions, including the frontal lobes, parietal lobes, caudate nucleus, and cerebellum bilaterally, and right olfactory cortex; (4) the patients with TAI presented altered long-distance connections in a functional subnetwork; (5) the nodal degree of the right inferior parietal lobule and nodal clustering coefficient of the right cerebellar hemisphere (VII) were negatively correlated with the MMSE score. These results also show that graph theory analysis is a powerful tool to examine the aberrant neural topology in patients with TAI and characterize the pathophysiology at the level of brain networks.

Large-scale brain networks typically show efficient economic small-world organization with short normalized path lengths and high normalized clustering coefficients, reaching an optimal balance between global integration and local specialization (Bassett & Bullmore, 2009; Bullmore & Sporns, 2009). In previous studies, a reduced small-world index was observed in patients with severe TBI (Nakamura et al., 2009); in contrast, Kuceyeski et al. (2016) reported a higher small-world index. However, our study showed that patients with TAI and HCs both had typical small-world attributes in brain functional networks, with no significant group differences in small-world metrics, including σ, γ, and λ, consistent with previous studies in patients with moderate-to-severe TBI (Caeyenberghs et al., 2012; Messe et al., 2013). The present findings suggest an overall preserved organization of the functional brain network in patients with TAI. However, how TAI affects small-world topology remains unclear with mixed results in prior studies. Besides, the small-worldness may also mistakenly identify a small-world topology in poorly segregated networks that are highly integrated, and should be a combination of different graph metrics capturing both integration and segregation (Caeyenberghs et al., 2017).

The loss of global integration is often interpreted as reduced efficiency of information processing in the networks. Altered topological architecture of functional networks in patients with TBI was found in previous studies(Han et al., 2016; Nomura et al., 2010; Pandit et al., 2013), revealing a reduction in the global integration of network efficiency interpreted as poor large-scale information transfer throughout the network (Rubinov & Sporns, 2010). However, patients with TAI in the current study showed no significant difference in E_glob_. The finding suggested that the capacity for information integration between distant brain regions and the efficiency of information dissemination in the global network were preserved, consistent with a previous study (Spielberg et al., 2015). This could be explained by the global brain network restoring effective functional connections via alternate structural pathways that circumvent the impaired white matter connections (Kuceyeski et al., 2016). Nevertheless, patients with TAI in our study exhibited a reduction in network efficiency with a lower E_loc_, predominantly related to short-range connections between adjacent nodes. Han et al. (2016) reported reduced global and local efficiencies in chronic TBI, whereas Caeyenberghs et al. (2012) found higher values of local efficiency in the TBI group. Such inconsistencies may be due to the heterogeneous characteristics of TBI and different methodologies. Our finding of reduced E_loc_ in patients with TAI could be attributed to inadequate axonal wiring or differences in metabolic running costs to provide parallel information compared to HCs.

The graph theoretical approach also allows the evaluation of regional characteristics of whole-brain functional networks using regional network measures, including nodal degree, nodal betweenness, nodal clustering coefficient, nodal efficiency, and nodal local efficiency. Several studies on TBI have shown alterations in nodal metrics in specific brain regions. For example, Pandit et al. (2013) showed a significant reduction in degree and betweenness centrality in the posterior cingulate cortex in TBI patients, whereas Caeyenberghs et al. (2012) reported higher betweenness centrality in the dorsal premotor cortex and dorsolateral prefrontal cortex in patients with moderate-to-severe TBI. The recent study observed decreased betweenness centrality, clustering coefficient, and local efficiency in several brain areas, including the fronto-parietal attention network (Kim et al., 2022).Our study showed higher nodal metrics values (nodal degree and nodal efficiency) in the left dorsolateral superior frontal gyrus in patients with TAI, as well as higher in nodal centralities (nodal betweenness, degree) and nodal efficiency in the medial superior frontal gyrus bilaterally, and higher nodal betweenness in the left superior orbital gyrus. These findings on basic measures and segregation suggest a higher functional connectivity in specific brain regions, which may reflect an adaptive mechanism to diminish injury-related symptom severity and maintain and/or stabilize neurocognitive performance (Hillary and Grafman, 2017). This modification may be attributed to numerous biophysical mechanisms, including hyperexcitability or disinhibition of functionally related networks (Fornito et al., 2015; Hillary et al., 2014). Hyperconnectivity is substantially universal in patients with TBI, regardless of the injury phase (acute, subacute, or chronic) and severity, replacing the traditional view of a transient process (Caeyenberghs et al., 2012; Iraji et al., 2016). The finding of increased functional connectivity in segregation after TAI is similar to the results of studies on healthy aging (Heitger et al., 2013) and some neurological diseases, such as brain tumors (Bartolomei et al., 2006; Bosma et al., 2008). Nonetheless, hyperconnectivity should not be automatically interpreted as supporting the compensation hypothesis in brain injuries; this concept is likely to be metabolically costly (Fornito et al., 2015), resulting in reduced adaptability to regulate the activity levels of network nodes. Caeyenberghs et al. (2012) demonstrated that patients with moderate-to-severe TBI who showed a higher connectivity degree presented a lower switching performance. The current finding suggests that the activity levels across multiple network nodes may show a higher level of synchronization in patients with TAI compared to HCs, in response to a damaged neurobiological substrate. In fact, volume loss and diffuse axonal injury are among the most common pathophysiologic sequelae of TBI (Benjamini et al., 2021; Bourke et al., 2022). The dorsolateral prefrontal cortex receives visual, somatosensory, and auditory information, and plays a central role in the cognitive control of motor behaviors (Miller & Cohen, 2001). Therefore, the higher nodal efficiencies in the dorsolateral prefrontal cortex in patients with TAI may indicate a less automatic movement generation. TBI most commonly disrupt the frontal system and interrupt the executive control processes (Hillary et al., 2002). Moreover, Alstott et al., (2009) demonstrated that a targeted injury of the frontal lobes induces a severe disruption of this network.

Reduced nodal efficiency values (including nodal degree, nodal clustering coefficient, and local efficiency) were observed in the caudate nucleus bilaterally in patients with TAI compared to HCs. Simoni et al. (2018) reported that patients with TBI showed a disruption of the functional connectivity between the caudate nucleus and several cortical regions, associated with cognitive impairments. Fagerholm et al. (2015) demonstrated that betweenness centrality and eigenvector centrality were reduced in the cingulate cortex and caudate due to the impact of a TAI on network connections. Furthermore, caudate activity during executive function tasks is associated with prefrontal measures of structural connectivity (Casey et al., 2007). Disruption of the fronto-caudate interactions may underpin the common cognitive impairments observed in patients with TBI. Interestingly, our NBS results showed significantly reduced long-distance functional connectivity between the caudate and cerebellum, especially the right caudate. This finding indicates the presence of pathway and interplay disturbances between the cerebellum and cerebrum due to damaged white matter connections between network nodes caused by a TAI. These alterations can disrupt the network function and lead to cognitive impairment.

Moreover, we observed higher nodal efficiency values (including nodal clustering coefficient, nodal efficiency, and nodal local efficiency) in the right cerebellar hemisphere (Crus 2, VII) and cerebellar vermis (4/5, 7). However, significant reductions in nodal efficiencies emerged in the left cerebellar hemisphere (VIII) and cerebellar vermis (10). These findings suggest that the cerebellar regional network is disrupted in patients with TAI. In addition, our NBS results revealed disrupted functional connectivity between the cerebellum and cerebrum, including reduced functional connectivity between the caudate and cerebellum, whereas higher functional connectivity between the olfactory cortex and cerebellum. Previous studies reported cerebellar atrophy in patients with TBI (Drijkoningen et al., 2015) and lower activation within the cerebellum (Yang et al., 2012). Our previous work also showed abnormalities in neural activity intensity and its temporal variability (Zhou et al., 2021) and decreased interhemispheric coordination in the cerebellum posterior lobes (Li et al, 2017). Moreover, our latest study revealed a disrupted functional connectivity of the striatal-cerebellar loop, whihc was strongly correlated with the prognosis of TAI patients (Xu et al., 2023). Further correlation analyses also showed that the nodal clustering coefficient of the right cerebellar hemisphere (VII) was negatively correlated with the MMSE score, suggesting a correlation with cognitive disorders in patients with TAI. Based on the above findings, we inferred that altered nodal efficiencies in the cerebellar network and disrupted functional connectivity between the cerebellum and cerebrum suggested the abnormality of cerebellar-cerebral connections, and might underlie the pathophysiology of the cognitive impairments seen in patients with TAI. However, the finding is needed more studies to confirm the reliability.

Compared to HCs, the TAI group observed significantly reduced nodal efficiency values (including nodal betweenness, degree, clustering coefficient, efficiency, and local efficiency) in the inferior parietal lobule, including the right angular gyrus and supramarginal gyrus bilaterally. The inferior parietal lobule is often involved in spatial perception, visuomotor integration, and sensory and memory circuitry. Additionally, this inferior parietal lobule is also key node of the posterior default network, which has been widely investigated and exhibited alterations in nodal activity within the network and in the intrinsic connectivity between networks (Sharp et al., 2014), and failure to deactivate the default network has been associated with poorer cognitive function in patients with TBI (Bonnelle et al., 2011). Furthermore, the NBS results showed reduced functional connectivity between the left supramarginal gyrus and the right inferior parietal lobule (angular gyrus), as well as between the right supramarginal gyrus and the right olfactory cortex. Meanwhile, the nodal degree of the right inferior parietal lobule had significant negative correlations with the MMSE score, speculating that may be involved in cognitive performance in TAI patients, However, the pathophysiology mechanism of the related cognitive impairments is not very clear. Our novel demonstration requires further study.

Prior studies have repeatedly demonstrated that individuals with TBI often present impairments in facial and vocal affect recognition and empathy (Babbage et al., 2011; Hopkins et al., 2002; Milders et al., 2003). Emotion recognition and empathy are key components of successful interpersonal interactions and relationships. Olfactory deficits are also common after TBI, affecting more than 56% of patients (Neumann et al., 2012). However, in practice, there is a lack of awareness regarding this deficit. Hopkins et al. (2002) found that patients with a closed-head injury who presented poorer emotion recognition and arousal responses to facial expressions compared to controls also had lower olfactory test scores. Similarly, Neumann et al. (2012) showed that olfactory deficits might be indicative of impaired affect recognition and reduced empathy after TBI. Our study showed a reduction in nodal local efficiency in the right olfactory cortex in patients with TAI. Furthermore, the NBS results showed reduced functional connectivity between the right olfactory cortex and the right supramarginal gyrus, and higher functional connectivity between the right olfactory cortex and cerebellar vermis. These findings suggest that the involvement of the olfactory cortex may be closely related to emotional processing impairments in patients with TAI.

The present study has several limitations. First, considering the heterogeneity of TBI, we selected a subgroup of relatively “pure” TAI patients among the 57 available; 29 patients were not examined due to larger local hemorrhages, and the final sample size was relatively small. We will stick to keep recruiting patients with TAI to conduct a large-sample in next work, further performing more precise subgroup analyses. Second, multiple comparison correction was eschewed as an exploratory analysis on topological properties in TAI patients, similarly reported altered graph-theory measures without correction for multiple comparison in previous studies (Chen et al., 2021; Hou et al., 2019; Shi et al., 2021). We will further improve the interpretability of statistical analysis by a large-sample study and consider the influence of different brain network segmentation templates in next work. Third, we only analyzed the differences in the functional brain networks between patients with TAI and HCs. Future research should examine the combination of functional and structural connectivity at the subject level. Fourth, this study was cross-sectional, and further longitudinal analyses are needed to investigate the predictive value of graph metrics.

## Conclusions

The brain networks of individuals with TAI undergo significant changes in topology, visible mainly in regional network measures, with no significant difference in small-world properties and global integration compared to HCs. These findings indicate that the graph theoretical analysis of fMRI data can provide useful information on the alteration of neural networks in patients with TAI. Aberrant topological attributes may be associated with cognitive impairment and might be potential biomarkers for predicting neurological dysfunctions, allowing further understanding of the neuropathological mechanism in patients with TAI.

## Funding 

This study was supported by the National Natural Science Foundation of China (Grants No. 81760307 and 82360341), the Natural Science Foundation Project of Jiangxi Province (Grants No. 20202BABL216038, 20232BAB216044), the Postgraduate Innovation Special Funding Project (YC2023-B066), and the Science and Technology Project of Jiangxi Health Committee (Grant No. 202110018).

### Supplementary Information

Below is the link to the electronic supplementary material.

Supplementary file1 (DOCX 24 KB)

## Data Availability

Not applicable.
