# Supplementary material for: Disrupted topological organization of functional brain networks in traumatic axonal injury
Source: Brain Imaging Behav. 2023 Dec 4;18(2):279–91. doi: 10.1007/s11682-023-00832-z (PMC11156726; doi:10.1007/s11682-023-00832-z)
Supplement: Supplementary file 1 — Supplementary file1 (DOCX 24 KB) [file 11682_2023_832_MOESM1_ESM.docx]

Supplementary material 1. Nodal centralities in the brain functional networks.

| Region | MNI | | | Nodal betweenness | | Nodal degree | | Nodal clustering coefficient | | Nodal efficiency | | Nodal Local efficiency | |
| --- | --- | --- | --- | --- | --- | --- | --- | --- | --- | --- | --- | --- | --- |
|  | X | Y | Z | t-value | p-value | t-value | p-value | t-value | p-value | t-value | p-value | t-value | p-value |
| TAI＞HCs |  |  |  |  |  |  |  |  |  |  |  |  |  |
| L Superior frontal gyrus | -18.45 | 34.81 | 42.20 | 1.81 | 0.08 | 2.95 | 0.005* | -0.16 | 0.87 | 2.71 | 0.009* | 0.47 | 0.64 |
| L Superior orbital gyrus | -16.56 | 47.32 | -13.31 | 2.10 | 0.041* | 1.79 | 0.08 | -0.93 | 0.35 | 1.59 | 0.12 | -0.50 | 0.62 |
| L Superior medial gyrus | -4.80 | 49.17 | 30.89 | 2.16 | 0.035* | 2.46 | 0.017* | -1.14 | 0.26 | 2.19 | 0.033* | -0.46 | 0.65 |
| R Superior medial gyrus | 9.10 | 50.84 | 30.22 | 2.03 | 0.048* | 2.35 | 0.022* | -1.45 | 0.15 | 2.09 | 0.041* | -0.88 | 0.38 |
| R Cerebellum (crus 2) | 32.06 | -69.02 | -39.95 | -0.67 | 0.51 | 0.97 | 0.34 | 2.10 | 0.040* | 0.98 | 0.33 | 2.13 | 0.038* |
| R Cerebellum (VII) | 33.14 | -63.18 | -48.46 | -0.60 | 0.55 | 0.35 | 0.73 | 2.46 | 0.017* | 0.54 | 0.59 | 1.78 | 0.08 |
| Cerebellar vermis (4/5) | 1.22 | -52.36 | -6.11 | -0.96 | 0.34 | 1.02 | 0.31 | 2.45 | 0.017* | 1.17 | 0.25 | 2.45 | 0.018* |
| Cerebellar vermis (7) | 1.15 | -71.93 | -25.14 | 1.40 | 0.17 | 2.00 | 0.05 | 1.51 | 0.14 | 2.04 | 0.046* | 1.82 | 0.07 |
| R Cerebellum (VI) | 24.69 | -58.32 | -23.65 | 3.10 | 0.003* | 2.21 | 0.03* | -2.26 | 0.03 | 1.87 | 0.07 | -2.39 | 0.02 |
| TAI＜HCs |  |  |  |  |  |  |  |  |  |  |  |  |  |
| R Olfactory cortex | 10.43 | 15.91 | -11.26 | 0.887 | 0.379 | 0.427 | 0.671 | -1.98 | 0.05 | -0.01 | 0.99 | -2.15 | 0.036* |
| L MCC | -5.48 | -14.92 | 41.57 | -1.30 | 0.20 | -2.00 | 0.05 | 1.69 | 0.10 | -2.38 | 0.021* | 1.24 | 0.22 |
| R Superior occipital gyrus | 24.29 | -80.85 | 30.59 | -2.43 | 0.019* | -1.56 | 0.13 | 0.92 | 0.36 | -1.66 | 0.10 | 0.30 | 0.77 |
| L Postcentral gyrus | -42.46 | -22.63 | 48.92 | -2.21 | 0.031* | -1.63 | 0.11 | 1.53 | 0.13 | -1.84 | 0.07 | 1.26 | 0.21 |
| R Inferior parietal Lobule | 46.46 | -46.29 | 49.54 | -2.33 | 0.023* | -2.50 | 0.015* | -0.19 | 0.85 | -2.73 | 0.008* | -1.35 | 0.18 |
| L Supramarginal Gyrus | -55.79 | -33.64 | 30.45 | -1.12 | 0.27 | -2.33 | 0.023* | -2.04 | 0.046* | -2.48 | 0.016* | -3.00 | 0.004* |
| R SupraMarginal Gyrus | 57.61 | -31.50 | 34.48 | -0.91 | 0.37 | -2.45 | 0.018* | -1.09 | 0.28 | -2.63 | 0.011* | -1.79 | 0.08 |
| L Caudate nucleus | -11.46 | 11.00 | 9.24 | -1.05 | 0.30 | -2.64 | 0.011* | -2.34 | 0.023* | -1.97 | 0.05 | -2.31 | 0.025* |
| R Caudate nucleus | 14.84 | 12.07 | 9.42 | -0.30 | 0.76 | -1.74 | 0.09 | -1.98 | 0.05 | -1.77 | 0.08 | -2.08 | 0.042* |
| L Cerebellum (VIII) | -25.75 | -54.52 | -47.68 | -2.30 | 0.025* | -0.91 | 0.37 | 0.94 | 0.35 | -0.63 | 0.53 | -0.08 | 0.93 |
| Cerebellar vermis (10) | 0.36 | -45.80 | -31.68 | -0.72 | 0.47 | -1.85 | 0.07 | -2.29 | 0.026* | -1.87 | 0.07 | -2.22 | 0.031* |
| R Cerebellum (VI) | 24.69 | -58.32 | -23.65 | 3.10 | 0.00 | 2.21 | 0.03 | -2.26 | 0.028* | 1.87 | 0.07 | -2.39 | 0.02* |

Patients with traumatic axonal injury(TAI) showed several alterations in nodal metrics in the brain functional network with significant between-group differences in at least one of the five nodal centralities. *p < 0.05, Uncorrected. Abbreviations: MNI, montreal neurological institute; R, right; L, left.
